# Dispersal of *Hyalomma rufipes* by migratory birds in northeastern Spain: Implications for Crimean-Congo haemorrhagic fever surveillance

**DOI:** 10.1016/j.onehlt.2026.101475

**Published:** 2026-06-10

**Authors:** Laura Carrera-Faja, David Bru, Carla Giupponi, Karine Huber, Carles Durà, Joan Castany, Vicente Ferrís Malonda, José Carmona Serrano, Xavier Fernandez Aguilar, Johan Espunyes, Laurence Vial, Oscar Cabezón

**Affiliations:** a Wildlife Conservation Medicine Research Group (WildCoM), Departament de Medicina i Cirurgia Animals, Universitat Autònoma de Barcelona (UAB), 08193 Bellaterra, Spain; b ASTRE, CIRAD, INRAE, Université de Montpellier, Montpellier, France; c CIRAD, UMR ASTRE, F-34398 Montpellier, France; d Estació Biològica del Montseny, Institut Català d'Ornitologia (ICO), Edifici Fontmartina, 08081 Fogars de Montclús, Spain; e Grup Au d'Ornitologia, 12001 Castelló, Spain; f IRTA. Programa de Sanitat Animal. Centre de Recerca en Sanitat Animal (CReSA). Campus de la Universitat Autònoma de Barcelona (UAB), 08193 Bellaterra, Catalonia, Spain; g Unitat mixta d'Investigació IRTA-UAB en Sanitat Animal. Centre de Recerca en Sanitat Animal (CReSA). Campus de la Universitat Autònoma de Barcelona (UAB), Bellaterra 08193, Catalonia, Spain; h Departament de Medi Ambient i Sostenibilitat, Ministeri de Medi Ambient, Agricultura i Ramaderia, Govern d'Andorra, AD500, Andorra

**Keywords:** Tick-borne pathogens, Mediterranean ecological region, Passeriformes, Population genetics, CCHFV

## Abstract

Migratory birds play a crucial role in the long-distance dispersal of ticks and tick-borne pathogens, contributing to the introduction of tick species and pathogens into new areas. Among those ticks, *Hyalomma rufipes* is a key concern due to its role as a vector of Crimean-Congo haemorrhagic fever virus (CCHFV) and its potential to establish in southern Europe. We assessed tick infestation, CCHFV presence, and both the probability of *H. rufipes* infestation and its genetic diversity in migratory birds captured at four key stopovers in northeastern Spain Mediterranean (NESM) ecological region during spring migration from 2022 to 2024. A total of 14,472 birds representing 84 species were examined, of which 0.5% carried ticks, primarily *H. rufipes* (77% of ticks), while all ticks tested negative for CCHFV. Statistical analyses revealed that ground- and shrub-foraging species, as well as birds wintering in open habitats and wetlands, had significantly higher probabilities of *H. rufipes* infestation. Genetic analyses of 88*H. rufipes* sequences showed high haplotype diversity with no clear population structure, indicating that ticks were acquired from multiple geographic origins, predominantly Central-West Africa. Some birds carried genetically distinct ticks simultaneously, which could facilitate pathogen transmission between ticks from different origins through co-feeding. Notably, some pre-Saharan migratory birds carried *H. rufipes*, suggesting potential local acquisition in North Africa, where the species has been previously reported although its establishment remains debated. These findings highlight the ongoing risk of introduction and possible establishment of *H. rufipes* in the NESM, with important implications for future surveillance of vector populations and tick-borne pathogens, including CCHFV, in Europe.

## Highlights

• Migratory birds in Northeastern Spain carry *Hyalomma rufipes* ticks from Africa.
• Bird species contribute unequally to the dispersal of CCHFV vectors in Europe.
• In *Passeriformes*, tick infestation depended on foraging behaviour and winter habitat.
• Multiple tick lineages on single birds suggest diverse infestation sources.
• Recurrent *Hyalomma rufipes* introductions via migratory birds may facilitate establishment in Europe.

## 1 Introduction

Among emerging infectious diseases, multi-host vector-borne diseases have gained importance over the last decades as their transmission dynamics are particularly sensitive to climate and environmental changes [1], [2]. Ticks are among the most important biological vectors of bacterial and viral pathogens, and they can be passively transported long distances by their hosts. As such, migratory birds can disperse tick-borne pathogens outside endemic areas and contribute to the introduction of tick species into new areas. Along their migratory routes between Africa and the Eurasian Palearctic, birds may encounter these arthropods not only at their departure sites but also during stopovers [3], [4]. Many studies investigating the ticks carried by birds during spring migration from African to European grounds revealed that sub-Saharan African hard ticks (Ixodidae) are found outside their native range on trans-Saharan migratory birds, primarily Passeriformes [5], [6], [7], [8]. The most common sub-Saharan hard tick species detected in migrant birds is *Hyalomma rufipes,* a key vector of Crimean-Congo haemorrhagic fever virus (CCHFV).

CCHFV is a tick-borne Bunyavirus within the genus *Orthonairovirus,* which can induce a haemorrhagic systemic disease in humans, with case-fatality rates ranging from 5% up to 40% [9]. Due to the severity of the disease, the absence of available vaccines, limited efficacy of antiviral specific treatments, and the potential for outbreaks, the World Health Organization (WHO) has identified CCHF as a priority disease for research and development [10]. CCHFV is endemic in several countries of Asia, Africa, the Middle East and south-eastern Europe, with a range similar to that of its main vector and reservoir, ticks of *Hyalomma* genus [9]. The natural cycle of CCHFV involves ticks from different stages that feed on several animal species, such as lagomorphs, rodents, birds or ungulates. *Hyalomma* ticks are considered both vectors and reservoirs of CCHFV, since they become infected for life and can transmit the virus trans-ovarially and trans-stadially [9].

CCHFV has recently emerged in south-western Europe. In Spain, CCHFV was first detected in 2010 in *Hyalomma lusitanicum* ticks from a red deer (*Cervus elaphus*) and, since 2016, 21 CCHF human cases have been diagnosed in western and central areas of the country [11], [12]. Viral strains identified in Spain showed high genetic variability, suggesting repeated introductions from different African and eastern Europe origins [13]. In the north-eastern Spain Mediterranean (NESM) ecological region, which differs from the Iberian sclerophyllous and semi-deciduous forest regions where CCHF cases occurred, this virus has never been detected despite the presence of tick species known to be competent vectors – *H. marginatum* and *H. lusitanicum*. However, CCHFV was recently detected in *Hyalomma marginatum* ticks from southern France [14], near the Spanish border and within the NESM ecological region. Moreover, two serological studies performed in wild ungulates from the NESM ecological region identified two foci of high CCHFV seropositivity in wild ungulates and described a long-term endemicity of the virus [15], [16]. These foci of CCHFV seropositivity in NESM are located near key stopovers for migratory birds from Africa, adding weight to the hypothesis of migratory birds having an important role in the introduction of CCHFV and its vectors of CCHFV into new areas.

Evidence of an active role of migratory birds in the dispersion of CCHFV-infected ticks is provided by studies performed in Italy, Turkey and Greece [17], [18], [19]. Moreover, CCHFV was detected in *Hyalomma* spp. ticks from trans-Saharan migrant passerine birds in Morocco, heading towards the Iberian Peninsula from sub-Saharan Africa [20]. Studies performed in Spain have failed to detect CCHFV in ticks from birds [21], [22], [23]. However, these studies were carried out more than 10 years ago, before the first CCHF cases were detected in Spain. In addition, the role of migratory birds in the introduction of *Hyalomma* spp. ticks and CCHFV in the NESM has never been assessed. Considering that two of the three Paleartic-African flyways connecting Europe with Africa - the Mediterranean / Black Sea flyway and the East Atlantic flyway - converge in the NESM ecoregion [24], it is crucial to closely monitor ticks and tick-borne pathogens on migratory birds reaching this region. Moreover, *H. rufipes* is the most common sub-Saharan tick detected on migratory birds at Mediterranean stopover sites in Europe, although it has not yet stablished permanent populations. Nevertheless, a recent study evaluating the environmental niche of *H. rufipes* indicated that the NESM ecological region provides suitable conditions for supporting permanent populations of this tick species [25]. This underscores the need for targeted surveillance of *H. rufipes* and its potential role in the introduction of CCHFV into the area.

The aim of this study was to evaluate the dispersion of ticks and CCHFV through migratory birds from Africa to NESM ecoregion in Spain. Moreover, this study aims to further characterize the genetic variability of *H. rufipes* using population genetic tools based on mitochondrial markers. These analyses are intended to explore patterns of genetic relatedness among detected sequences and infer its geographic origin. In addition, the study examines the potential role of different migratory bird species, with diverse ecological traits, in the introduction of *H. rufipes* into the NESM.

## 2 Materials and methods

### 2.1 Tick collection

**Fig. 1:**
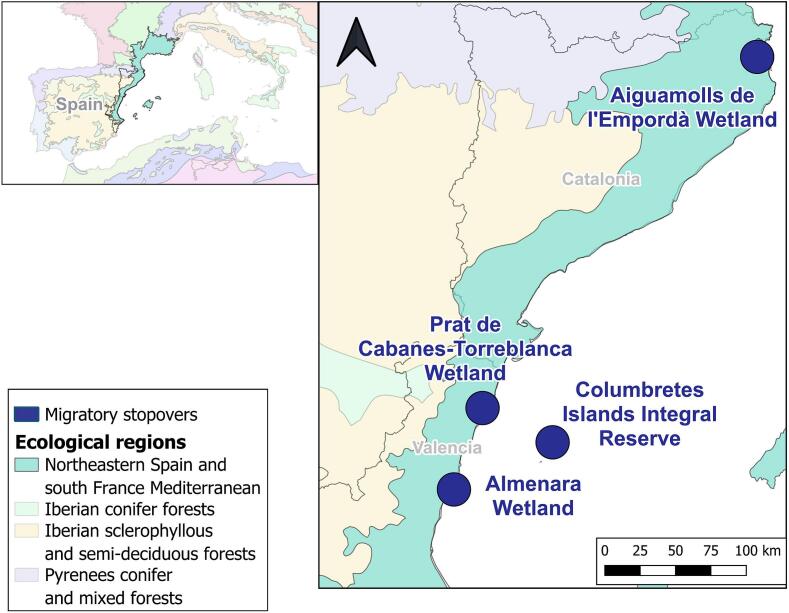
Bird sampling sites in the Northeastern Spain Mediterranean (NESM) ecological region (Catalonia and Valencia).

Bird sampling was performed from March to the beginning of June during the years 2022–2024, when the prenuptial migration northward occurs. Sampling activities were carried out in four key migratory stopovers within the NESM ecological region: Empordà Wetland, Prat de Cabanes-Torreblanca Wetland, Almenara Wetland and Columbretes Islands Integral Reserve **(**Fig. 1**)**. This ecological region, which comprises the Autonomous Communities of Catalonia and Valencia, is characterized by very hot and dry summers, and relatively temperate mild and humid winters. Vegetation is dominated by sclerophyll forests, dense shrublands and Mediterranean pine woodlands.

Bird captures were performed using mist nets and were always conducted by licensed bird ringers. Individuals were uniquely and permanently ringed on first capture, and biometrical measures were taken, following the European Union for Bird Ringing guidelines (https://www.euring.org). Ticks were removed using stainless-steel tweezers and preserved in RNAlater Stabilization Solution (Invitrogen, ThermoFisher Scientific, US) until the end of the ringing season.

### 2.2 Tick identification

Ticks were preliminarily identified to genus-level using magnifying lenses and taxonomic keys [26], [27]. Because most of the specimens collected were immature stages, ticks were molecularly analysed for species-level determination. Ticks were washed twice with 70% ethanol and once with sterile phosphate-buffered saline (PBS), and their legs were removed for genomic DNA extraction. Mechanical homogenization of the tick legs was performed by adding three 2.8 mm stainless steel beads (Bertin Technologies, Montigny-le-Bretonneux, France) and using Tissue-lyser (Qiagen, Hilden, Germany) for 2 min at 30 Hz. Nucleic acids were extracted using NucleoMag VET kit (Macherey-Nagel, Düren, Germany) and the automated KingFisher Flex Purification System (ThermoFisher Scientific, Waltham, USA), and eluted in a final volume of 70 μl.

For tick species identification and phylogenetic characterization, two primer pairs were used to amplify the COI gene; TY-J-1449 / C1-N-2312 and Cox1F / Cox1R [28], [29]. PCR amplification reactions were performed in a total volume of 40 μl. PCR mix consisted of FailSafe™ PCR 2× PreMix C, FailSafe™ Taq (1.25 U), the primers (10 pmol each) and the genomic DNA (3 μl). PCR amplification conditions consisted of an initial denaturation step at 95 °C for 5 min followed by ten cycles of 92 °C for 1 min; 42 °C for 40 s; 72 °C for 1 min 30 s; 32 cycles of 92 °C for 1 min; 46 °C; 72 °C for 1 min 30; and a final extension step at 72 °C for 7 min. Gel migration was performed to ensure the quality of the amplification and to check the size of the amplified DNA fragments. The PCR products were sent for sequencing by AZENTA - GENEWIZ using the Sanger method. Since the COI gene was amplified using two different primer pairs for obtaining a larger sequence, only one primer from each pair was used for sequencing (Cox1F and C1-N-2312) [30]. COI DNA sequences were deposited in GenBank under Accession Number PZ371339-PZ371348, PZ380225-PZ380306 and PZ381163-PZ381173.

Sequences were assembled with Geneious v.6.0.5 (Biomatters, Aukland, New Zealand), aligned with MUSCLE algorithm and were identified at the species level using the Basic Local Alignment Search Tool (BLAST) software. A sequence identity of over 99% between the sequence and the reference was used to confirm sample identification. COI DNA sequences of four *Hyalomma* spp. and three *Ixodes* spp. were also obtained from GenBank to validate and cross-check the species identifications across studies.

### 2.3 CCHFV detection

For viral RNA extraction, 350 μl of AVL buffer (Qiagen, Hilden, Germany) were added to each tick sample and mechanically disrupted using plastic tissue homogenisers. RNA was extracted from 140 μl of the tick AVL-supernatant using the QIAamp Viral RNA Mini Kit (Qiagen, Hilden, Germany), following the manufacturer's instructions. The extracted RNA was eluted in 50 μl of RNAse-free water and stored at −80 °C prior to molecular analysis.

We used a one-step multiplex real-time RT-qPCR developed by Sas et al. 2018 [31] to detect the RNA of the CCHFV S segment. The RT-qPCR was performed using the QuantiTect Probe RT-PCR Kit (Qiagen, Hilden, Germany). Both positive and negative controls were included in each PCR run to validate the results. Positive controls consisted of synthetic RNA/DNA from known CCHFV strains (Genotype II DR Congo and Genotype V Kosovo), as detailed in Sas et al. [30], while negative controls consisted of sterile water.

### 2.4 *Associations between bird species' ecological traits and* H. rufipes

Binomial logistic regressions were fitted to assess if ecological traits of migratory bird species influence the probability of carrying *H. rufipes*. Because in our data some categorical combinations had low sample sizes and the standard maximum likelihood binomial logistic regression showed quasi-separation (infinite estimates), a bias-reduced binomial logistic regression (‘brglm2’ package) was used, which produces finite, bias-reduced estimates. The response variable was the number of birds infested with ticks, modelled as a binomial outcome, considering the count of infested birds and non-infested birds for each species. As predictor variables, bird species were classified by i) order, ii) migration distance (long, medium and resident/short), iii) foraging behaviour (aerial; shrubs and trees; ground), iv) predominant wintering habitat (wetland – i.e. marshes, swamps, river deltas, coastal lagoons; open habitat – i.e. grasslands, savannas, stepped and agricultural fields; forest and dense shrublands), v) type of migration (resident; pre-Saharan; trans-Saharan; partial migrant) and vi) wintering region (southern Europe; sub-Saharan Africa; North Africa). To avoid collinearity, Variance Inflation Factors (VIFs) of the six variables were tested using ‘performance’ package and those with VIF < 5 were selected. Model selection was performed using the Akaike Information Criterion corrected for small sample sizes (AICc) from the ‘MuMIn’ package. Models including predictors with clear ecological relevance were ranked according to their AICc values, and all models within ΔAICc ≤2 were selected. Predictor's significance was assessed though z-values and associated *p*-values, and the results were interpreted in terms of odds ratios. A p-value of less than 0.05 was considered statistically significant. Statistical analyses were carried out using R software version 4.4.1. [32].

### 2.5 Hyalomma rufipes *genetic diversity and structure*

DnaSP 6.12.03 [33] was used to assess genetic diversity between *H. rufipes* populations using the following statistics: the number of haplotypes (h), haplotypic diversity (Hd), nucleotide diversity (π) and the average number of nucleotide differences (k). To infer genealogical relationships between *H. rufipes* populations, migratory stopovers and bird host species, we constructed a haplotype network using the median-joining network method [34] using NETWORK 4.6.1.2 [35]. Genetic structure was assessed using a Bayesian clustering method implemented in the RhierBAPS 1.1.3 package [36], using 100 replicate runs., with a maximum number of populations (K) set to four, corresponding to the sampling sites. In addition, in order to explore the possible origin of *H. rufipes* specimens, a phylogenetic tree including COI sequences of *H. rufipes* collected in different African countries available in Genbank was inferred using IQ-TREE 2.4.0. The best-fit substitution models and partitioning scheme were automatically selected using ModelFinder with partition merging (MFP + MERGE), and branch support was assessed by 1000 ultrafast bootstrap replicates.

## 3 Results

### 3.1 Bird and tick species identification

A total of 14,472 birds of 84 different species were captured during the springs of 2022–2024. Most of the birds were trans-Saharan migrants (67.6%, 9781/14,472) followed by pre-Saharan migrants (21.75%, 3148/14,472). The most commonly captured bird was *Acrocephalus scirpaceus* (3807/14,472). A total of 73 birds from 19 different species presented ticks (0.5%) of which 77.33% were trans-Saharan migrants (58/73). 124 ticks were recovered from these birds, giving an average of 1.7 ticks per bird. The summary of bird species captured by site and year can be found in Supplementary Table 1.

Five tick species were detected, being *H. rufipes* the most frequent (96/124), followed by *Ixodes frontalis* (13/124), *H. marginatum* (3/124), *I. festai* (6/124) and *I. ricinus* (1/124) (Table 1). Five ticks (four *Ixodes* spp. and one *Hyalomma* spp.) could not be identified to the species level because no PCR amplicon was obtained. All the ticks were immature stages (larvae and nymphs) except for 8 females of *Ixodes* spp. (Table 1).

**Table 1:** Summary of ticks collected from bird species captured during the spring migration in the Northeastern Spain Mediterranean (NESM) ecoregion (2022–2024).

	Total ticks (total parasitized birds)							
Bird species	*Hyalomma* *marginatum*	*Hyalomma* *rufipes*	*Ixodes* *frontalis*	*Ixodes* *ricinus*	*Ixodes festai*	*Ixodes* sp.	*Hyalomma* sp.	Total
*Acrocephalus arundinaceus*		6 (4)						**6 (4)**
*Acrocephalus schoenobaenus*		7 (1)						**7 (1)**
*Acrocephalus scirpaceus*	2 (2)	53 (27)					1 (1)	**56 (30)**
*Anthus trivialis*		3 (1)						**3 (1)**
*Cettia* *cetti*			1 (1)					**1 (1)**
*Emberiza schoeniclus*						1 (1)		**1(1)**
*Erithacus rubecula*		1 (1)	5 (4)			3 (2)		**9 (7)**
*Lanius* *senator*		3 (2)						**3 (2)**
*Luscinia megarhynchos*	1 (1)		1 (1)					**2 (2)**
*Luscinia svecica*		4 (1)						**4 (1)**
*Motacilla* *flava*		4 (4)		1 (1)				**5 (5)**
*Phoenicurus phoenicurus*		7 (6)						**7 (6)**
*Phylloscopus collybita*			1 (1)					**1 (1)**
*Phylloscopus trochilus*			1 (1)					**1 (1)**
*Prunella modularis*					5 (2)			**5 (2)**
*Sylvia* *atricapilla*			1 (1)					**1 (1)**
*Sylvia* *communis*		8 (5)						**8 (5)**
*Turdus* *merula*					1 (1)			**1 (1)**
*Turdus philomelos*			3 (1)					**3 (1)**
Total	**3 (3)**	**96 (52)**	**13 (10)**	**1 (1)**	**6 (3)**	**4 (3)**	**1 (1)**	**124 (73)**

### 3.2 CCHFV molecular detection

All the ticks (*n* = 124) tested negative to the CCHFV RT-qPCR.

### 3.3 Associations between bird species' ecological traits and *H. rufipes*

Because only species within the *Passeriformes* order were infested with ticks, statistical analyses were carried out only in this order. The most parsimonious model was a bias-reduced binomial logistic regression including the variables ‘Foraging behaviour’, ‘Type of migration’ and ‘Wintering habitat’. All alternative models showed substantially lower support (ΔAICc >2). Infestation prevalence varied significantly with foraging behaviour and winter habitat. Species that forage on the ground (OR = 55.93, 95% CI: 3.20–977.37, *p* = 0.0058) or in shrubs and trees (OR = 58.57, 95% CI: 2.91–1179.10, *p* = 0.0079) had 55.93- and 977.37-times higher infestation probabilities compared to aerial foragers. Winter habitat was also important: species wintering in open habitats (OR = 8.08, 95% CI: 1.97–33.21, *p* = 0.0038) and wetlands (OR = 2.27, 95% CI: 1.17–4.42, *p* = 0.0154) showed higher odds of infestation than species wintering in forests/shrubs.

**Fig. 2:**
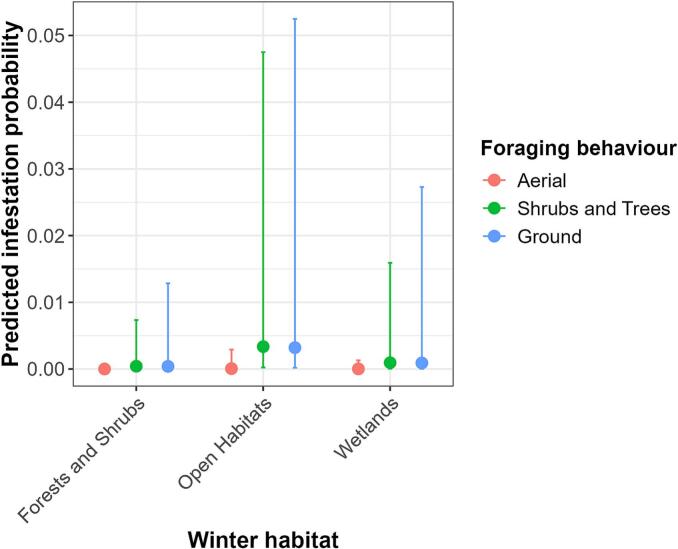
Predicted *H. rufipes* infestation probability (±95% CI) across winter habitats for each foraging strategy in passerine bird species, based on the bias-reduced binomial model.

Although Type of Migration was not significant (*p* > 0.05), it was retained in the model because its presence enhanced its explanatory capacity (higher McFadden R^2^ and lower AICc). Overall model fit was good (AICc = 100.5, McFadden's *R*^*2*^ = 0.40) (Table 2**,** Fig. 2).

**Table 2:** Odds ratios (95% CI) showing the effect of foraging behaviour, type of migration, and wintering habitat on the probability of *Hyalomma rufipes* infestation in *Passeriformes*. Estimates are from a bias-reduced binomial logistic regression (brglm2). The asterisk (*) represents statistically significant *p-*values (<0.05).

Model	ForagingBehaviour + TypeMigration + WinterHabitat	
Predictor	Odds ratio (95% CI)	*p*-value
Foraging behaviour		
Aerial	Reference	
Ground	55.93 (3.20–977.37)	0.0058*
Shrubs & Trees	58.57 (2.91–1179.10)	0.0079*
Type of migration		
Partial migrant	Reference	
Pre-Saharan	1.63 (0.07–38.41)	0.76
Resident	0.31 (0.01–13.87)	0.55
Trans-Saharan	7.77 (0.46–130.82)	0.15
Winter habitat		
Forests & Shrubs	Reference	
Open habitats	8.08 (1.97–33.21)	0.0038*
Wetlands	2.27 (1.17–4.42)	0.0154*
AICc	100.5	
McFadden R^2^	0.40

### 3.4 *Hyalomma rufipes* genetic diversity and structure

**Fig. 3:**
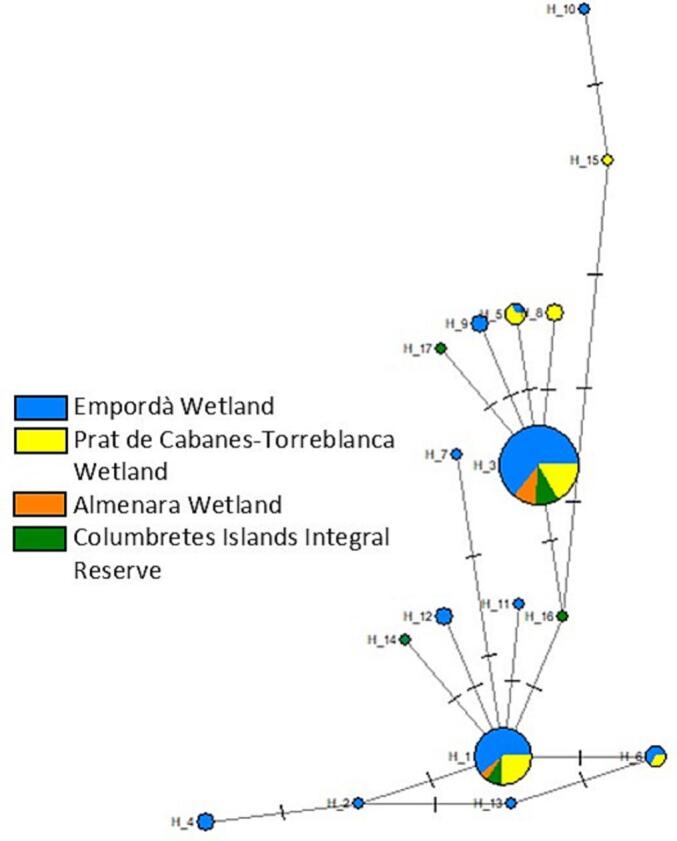
Median-joining haplotype networks for *H. rufipes* COI gene. The size of the circles is proportional to the number of individuals. The colours represent the migratory stopovers where the ticks were retrieved from birds. The length of the branches separating haplotypes is proportional to the number of mutational steps.

After sequence cleaning, assembly, and alignment, 88 sequences of *H. rufipes* with an edited length of 733 bp were used to assess genetic diversity. The population showed high genetic diversity, comprising 17 different haplotypes and a haplotype diversity of 0.706. The most abundant haplotypes were 3 and 1. The haplotype network (Fig. 3) was centred on these two main haplotypes, with most of the others linked to them, only differing by 2 or 3 single-nucleotide polymorphisms (SNPs). Fewer haplotypes showed higher genetic distance from the main haplotypes such as haplotype 10, which differed by 7 SNPs from haplotype 1. There was no evident pattern between the haplotypes and the migration stopover (Fig. 3) or the bird species whose ticks were parasitizing. Interestingly, two or three different *H. rufipes* haplotypes were found on bird individuals (*n* = 6) that were parasitized by multiple ticks, suggesting multiple infestation events and sources, or high genetic diversity at the site where they became infested. Bayesian analysis of population structure did not reveal any clear patterns of genetic structure or groupings. No remarkable clustering of individuals was visually correlated with migratory stopovers or bird species ecological traits.

**Fig. 4:**
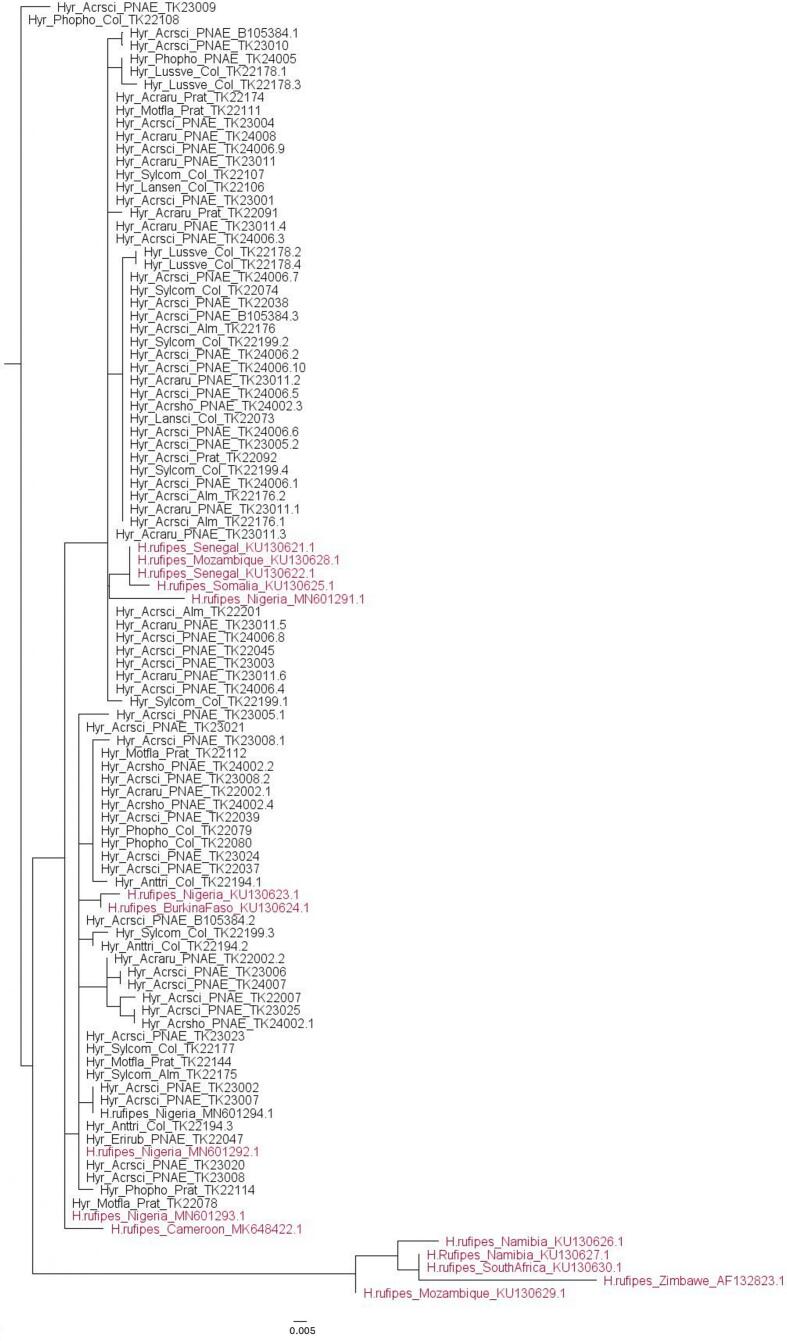
Maximum likelihood phylogenetic tree of *H. rufipes* based on the COI assembled gene sequences. Sequences from this study are in black while *H. rufipes* sequences from different African countries are shown in red. In each row, bird species with abbreviated six-letter scientific codes (three letters of the genus + three letters of the species), and the migratory stopover (PNAE = Empordà, Prat = Prat de Cabanes – Torreblanca, Alm = Marjal d'Almenara, Col = Columbretes Island) or country of origin are indicated. (For interpretation of the references to colour in this figure legend, the reader is referred to the web version of this article.)

In the *H. rufipes* phylogenetic tree, ticks from our study clustered with reference sequences of Central-West Africa (Burkina Faso, Senegal, Nigeria, Cameroon), whereas none clustered with south African countries (South Africa, Zimbabwe, Namibia) (Fig. 4).

## 4 Discussion

This study provides the first assessment of tick infestation and CCHFV in migratory birds within the NESM ecoregion. The percentage of birds infested with *Hyalomma* spp. was lower (0.42%) than studies performed in other Mediterranean regions [5], [20], [37]. This difference can be explained by the year-to-year differences on tick abundance or the higher latitude of our study area, consistent with other studies that observed a decline in *Hyalomma* spp. infestation rate as latitude increases [21]. Among the major CCHFV vector species (*Hyalomma* spp.), *H. rufipes* was the most frequently tick species detected, with *H. marginatum* occurring at a substantially lower frequency. Our findings are consistent with reports from other Mediterranean locations, where *H. rufipes* has been frequently detected on migrant birds [5], [6], [8]. Similar studies in other areas of Spain had not reported *H. rufipes*. However, they were either carried out outside Mediterranean regions [22] or more than 10 years ago without performing molecular identification, which is crucial for discerning between *H. rufipes* and *H. marginatum* immature stages [21], [23]. In fact, *H. rufipes* was considered a subspecies of *H. marginatum* until very recently, and molecular analyses are essential to distinguish these two closely related species. Therefore, to the best of our knowledge, this study represents the first confirmation of *H. rufipes* parasitizing migratory birds during their stopover in the Iberian Peninsula [38].

Although no positive samples for CCHFV were detected in this study, *H. rufipes* is a competent vector of this pathogen [9]. Indeed, this tick species has been found to have the highest CCHFV positivity proportion among *Hyalomma* species in Mauritania (5.7%) [39] and has also been detected carrying the virus in other African countries such as Senegal and Ghana [[40], [41]]. Therefore, the absence of detection may be attributable to the low probability of finding infected ticks in limited sample sizes. As the introduction and the predicted establishment of *H. rufipes* in Europe progresses [25], it is crucial to monitor not only tick distributions but also their potential involvement in the epidemiology of CCHFV and other pathogens such as Alkhurma haemorrhagic fever virus [42] and exotic *Rickettsia* spp. [7], [37]. In addition, the interaction between *H. rufipes* and autochthonous tick species from the NESM ecological region (e.g., *H. marginatum*, *H. lusitanicum*) may have unexpected implications for pathogen transmission dynamics.

*H. rufipes* is a two-host species typically associated with arid and semi-arid environments of sub-Saharan Africa. Its ecology, characterized by adaptation to high temperatures, low humidity and open habitats [26], supports its presence on migratory birds wintering in such environments. This ecological association was reflected in our statistical model, which identified open wintering habitats and wetlands, showing a weaker effect, as significant predictors of *H. rufipes* infestation. Expectedly, different foraging behaviours had a strong effect on the probability of *H. rufipes* infestation. Instead of passively questing on high vegetation*, H. rufipes* ticks detect the host by CO_2_ and movement detection, remaining more time on the soil surface where their senses are most efficient. Therefore, birds that forage on the ground or in shrubs experience much higher encounter probability with host-seeking *H. rufipes*. As *H. rufipes* known distribution is sub-Saharan Africa, we expected to find that Trans-Saharan migrators would have higher odds of being infested by this tick species. However, the type of migration did not show statistical significance in our model. In fact, we detected *H. rufipes* on two bird species that do not undertake trans-Saharan migration (*Luscinia svecica* and *Erithacus rubecula*), which may suggest the possibility of local acquisition in North Africa. To date, there are scattered reports of this tick species in North African countries [[43], [44], [45]], and it was believed to be the consequence of introduction via migratory birds. However, the infestation of pre-Saharan birds with immature stages of this species suggests that *H. rufipes* populations may already be established in North Africa. This observation is particularly relevant considering recent ecological niche models predicting the potential for *H. rufipes* to expand its distribution northwards under current and future climate scenarios [25]. Moreover, the reports of adults of *H. rufipes* moulting from nymphs in Europe have increased in recent years [[46], [47]]. However, the potential establishment of long-lasting populations in non-endemic European regions remains a subject of debate. While sporadic overwintering and reproduction have been noted in Central Europe and Russia, evidence suggests these may be short-lived events, possibly hindered by ecological factors such as high humidity [38]. Although the introduction of CCHFV-positive nymphs by migratory birds is a known phenomenon, the direct risk of virus transmission in non-endemic countries should be interpreted with caution. Current evidence lacks reports of CCHFV positive *H. rufipes* adults or autochthonous cases in these areas. Furthermore, significant gaps remain regarding the transstadial persistence of CCHFV in *H. rufipes* ticks that are not reinfected by viraemic hosts [48]. Therefore, additional data on transstadial transmission and survival are essential to fully evaluate their epidemiological relevance.

However, in contrast to the low habitat suitability of Central Europe where *H. rufipes* populations may struggle to persist, the semi-arid conditions and lower humidity of the Mediterranean basin offer a more suitable environment for its lifecycle [25]. Given the annual introduction of *H. rufipes* by migratory birds and the environmental suitability of the NESM ecoregion for sustaining permanent populations, our findings emphasize an increasing need for targeted surveillance of this tick species in the area, while maintaining a cautious interpretation of the immediate CCHFV transmission risk. Such surveillance should adopt a multi-disciplinary One Health approach, integrating ornithological expertise with wildlife veterinarians involved in avian disease surveillance, climate scientists and public health officials. Furthermore, expanding capacity through citizen science networks—such as the collection of deceased birds by informed local groups—could provide critical insights into the distribution and phenology of avian-associated tick-borne diseases [49].

Phylogenetic analyses did not reveal a clear genetic population structure of *H. rufipes*. However, most of the *H. rufipes* sequences clustered with sequences from ticks obtained in Sub-Saharan West Africa, aligning with the fact that most of the migratory birds captured in the NESM ecoregion stopovers come from this region [50]. In contrast, ticks from this study were genetically distant from *H. rufipes* collected in south African countries. As *H. rufipes* feeds on the same hosts during larval and nymphal stages and this period takes from two to three weeks [26] and up to 28 days under laboratory conditions [51], it is unlikely that ticks acquired in southern Africa would remain attached to the bird by the time they reach the NESM region.

Interestingly, genetically distinct *H. rufipes* lineages were found on individuals infested by multiple ticks, suggesting diverse sources of infestation. This may reflect acquisition from different maternal lineages within the same stopover site or cumulative infestations during migration. Since ticks infesting birds are usually found on head regions to avoid being removed by the host [52], ticks acquired from different origins would likewise aggregate together, and this could facilitate pathogen transmission between ticks through co-feeding, in which ticks in close proximity can transmit pathogens independently of systemic infection in the bird [53]. Given that birds are generally considered poor amplifiers of CCHFV with low or absent viremia [48], co-feeding transmission may represent a particularly relevant mechanism for this virus dissemination during migration. However, the role of co-feeding in pathogen transmission and maintenance during bird migration warrants further investigation.

## 5 Conclusion

Migratory birds are playing an important role in the dispersal of *H. rufipes* and this study provides further evidence of the current risk introduction and establishment of *H. rufipes* in the NESM ecological region. The ecological patterns observed, combined with genetic data, suggest that birds may acquire ticks from multiple origins, with possible implications for pathogen transmission via co-feeding. Although CCHFV was not detected in this study, the role of *H. rufipes* as a vector of high-impact zoonoses underscores the urgent need for continued surveillance and research in both vector and disease ecology along migratory stopovers.

## CRediT authorship contribution statement

**Laura Carrera-Faja:** Writing – original draft, Methodology, Investigation, Formal analysis, Data curation, Conceptualization. **David Bru:** Methodology, Formal analysis. **Carla Giupponi:** Visualization, Software, Formal analysis. **Karine Huber:** Writing – review & editing, Supervision, Formal analysis. **Carles Durà:** Methodology, Investigation. **Joan Castany:** Methodology, Investigation. **Vicente Ferrís Malonda:** Methodology. **José Carmona Serrano:** Methodology. **Xavier Fernandez Aguilar:** Writing – review & editing, Investigation, Formal analysis. **Johan Espunyes:** Writing – review & editing, Supervision. **Laurence Vial:** Writing – review & editing, Supervision. **Oscar Cabezón:** Writing – review & editing, Supervision, Resources.

## Funding

Laura Carrera-Faja was funded through the 2022 FI Scholarship, *Departament de Recerca i Universitats, Generalitat de Catalunya*, Spain (FI_B 00723). The present study was funded by Plan Estatal de Investigación Científica, Técnica y de Innovación, Agencia Estatal de Investigación, Ministerio de Ciencia e Innovación, Gobierno de España. Proyectos de generación de conocimiento: PID2024-158857OB-C21 and by the Agència de Gestió d'Ajuts Universitaris i de Recerca, Departament de Recerca i Universitats, Generalitat de Catalunya. Projectes de recerca per a la mitigació i adaptació al canvi climàtic: 2023CLIMA00103. This work was also funded by the French Ministry of Agriculture-General Directorate for Food (DGAL, grant agreement: SPA17 number 0079-E). Xavier Fernandez Aguilar acknowledges support under a Ramón y Cajal contract (RYC2022‑036927‑I), funded by the Agencia Estatal de Investigación (Spanish Ministry of Science, Innovation and Universities).

## Declaration of competing interest

The authors declare that they have no known competing financial interests or personal relationships that could have appeared to influence the work reported in this paper.

## Data Availability

Data will be made available on request.

## Appendix A Supplementary data

Supplementary material. Summary of bird species captured by site and year and the number of birds infested with at least one tick.
